# Gynecomastia during imatinib mesylate treatment for gastrointestinal stromal tumor: a rare adverse event

**DOI:** 10.1186/1471-230X-11-116

**Published:** 2011-11-02

**Authors:** HeLi Liu, GuoQing Liao, ZhongShu Yan

**Affiliations:** 1 Department of Gastrointestinal Surgery, Xiangya Hospital, Central South University, Changsha 410008, China

## Abstract

**Background:**

Imatinib mesylate has been the standard therapeutic treatment for chronic myeloid leukemia, advanced and metastatic gastrointestinal stromal tumor (GIST). It is well tolerated with mild adverse effects. Gynecomastia development during the course of treatment has been rarely reported.

**Methods:**

Ninety-eight patients with advanced or recurrent GIST were treated with imatinib mesylate. Among the fifty-seven male patients six developed gynecomastia during the treatment. The lesions were confirmed by sonography. Sex hormone levels were determined in six patients with and without the presence of gynecomastia respectively. The patients with gynecomatia were treated with tamoxifene and the sex hormones were assayed before and after tamoxifene treatment.

**Results:**

In patients with gynecomastia the lump underneath the bilateral nipples was 2.5 to 5 centimeters in diameter. Their serum free testosterone levels ranged between 356.61 and 574.60 ng/dl with a mean ± SD of 408.64 ± 82.06 ng/dl (95% CI 343.03~474.25 ng/dl), which is within the normal range. The level of serum estradiol was 42.89 ± 16.54 pg/ml (95% CI 29.66~56.12 pg/ml). Three patients had higher levels (43.79~71.21 pg/ml) and the others' were within normal range of 27.00~34.91 pg/ml. Six patients without the development of gynecomastia had normal free testosterone. One patient died because of large tumor burden. The sex hormones had no significant changes before and after tamoxifene treatment.(*P *> 0.05)

**Conclusions:**

Testosterone levels were not decreased in the six GIST patients with gynecomastia. Three patients had increased serum estradiol level which suggests that imbalance of sex hormones may be the cause of gynecomastia during treatment with imatinib mesylate.

## Background

The substantial efficacy of imatinib mesylate in the treatment of advanced and recurrent gastrointestinal tumors (GISTs) has been confirmed by clinical trials in the past decade. It has been considered as the standard treatment for GIST and chronic myeloid leukemia(CML) worldwide. Imatinib mesylate is well tolerated with only mild adverse effects, such as edema, nausea, vomiting, diarrhea, skin rash, fatigue, cramps and arthalgia, etc [1]. In general, mild degree of anemia and neutropenia are common when given a standard dosage, but they are easily controlled and no discontinuation of therapy is needed. Gynecomastia is an uncommon manifestation during the course of imatinib mesylate treatment. So far, there have been only two reports about this event [2,3]. Recently there was a report about the relationship between Dasatinib and gynecomastia [4]. The present study reported our experience in a single institution in China.

## Methods

From January 2005 to December 2010, 98 consecutive patients were treated for advanced or recurrent GIST in our department. They included 57 male patients, six of them developed gynecomastia during imatinib mesylate treatment. The lesions were confirmed by clinical examination and sonography. The median age was 62.0 years (range 35 ~76). The median duration of imatinib mesylate intake was 8.0 months (range 5~20 months). Imatinib mesylate 400 mg daily was given in one dose. All patients had prior surgery for GIST and had recurrence or metastasis of their diseases. Two patients had disseminated intra-peritoneal tumors and four patients had liver metastases. Sex hormone levels were measured in six patients with and without the presence of gynecomastia respectively. Written informed consent was obtained from all patients, and the protocol for this study was approved by the Ethics Committee of Xiangya Hospital, CSU (No.KY2010-32).

Quantitative values were presented as mean ± SD. Student's t test was used to compare the differences of sex hormones before and after tamoxifene treatment. Statistical analyses were performed using the SPSS software (version 13.0, Chicago, USA). All tests were two-tailed and differences were assumed as significant at *P *values of less than 0.05.

## Results

**Figure 1:**
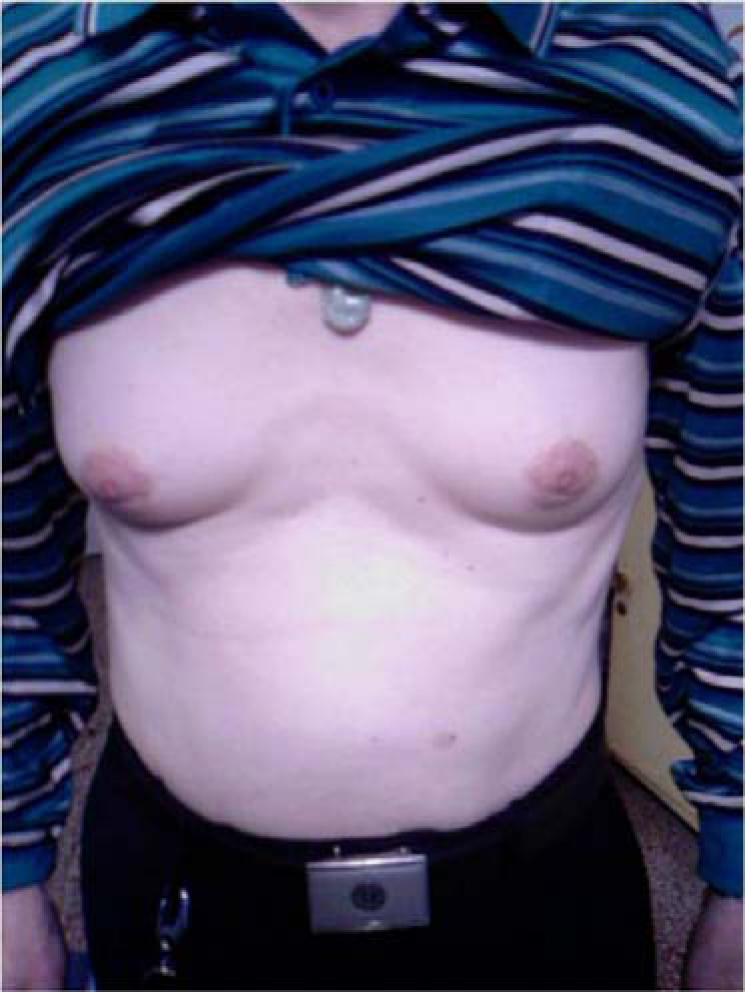
**54 year old man developed gynecomastia after receiving imatinib for five months**.

All six patients complained of painful lump under bilateral nipples during monthly clinical evaluation.On examination, the lumps were 2.5 to 5 centimeters in diameter with bilateral occurrence (Figure 1). Only one patient (76 years old) reported decrease in sexual activity but without impotence. Laboratory studies indicated that these six gynecomastia patients' serum free testosterone ranged from 356.61 to 574.60 ng/dl with a mean ± SD of 408.64 ± 82.06 ng/dl (95% CI 343.03~474.25 ng/dl), which is within the normal range (our laboratory normal value was 241~847 ng/dl). The mean level of serum estradiol measured was 42.89 ± 16.54 pg/ml (95% CI 29.66~56.12 pg/ml). Three patients had higher levels (43.79~71.21 pg/ml) and the others' were within normal range, 27.00~34.91 pg/ml (our laboratory normal value was 11.6~41.2 pg/ml). Blood samples taken from six randomly selected patients who do not have gynecomastia showed normal results of free testosterone, mean ± SD of 424.96 ± 94.37 ng/dl (95% CI 349.18~500.20 ng/dl). Liver function tests were normal in five of the six patients. The another patient suffered from chronic hepatitis B had an increase in serum transaminase and total bilirubin. He was referred to the hepatologist for further care. His serum free testosterone was normal but estradiol was higher than normal range with a reading of 71.21 pg/ml. Imatinib mesylate treatment was continued and no dosage adjustments were done. Tamoxifene citrate 20 mg daily was given to all patients with gynecomastia excluding the one with hepatitis B, the duration is 8-27 weeks. The sex hormones of the patients with gynecomastia were assayed before and after tamoxifene treatment, as showed in Table 1. All sex hormones had no significant changes before and after tamoxifene treatment(*P *> 0.05).

**Table 1:** Sex hormones of gynecomastia patients before and after tamoxifene treatment

Gynecomatia	Age	FSH (IU/L)*		LH (IU/L)#		Freet estosterone (ng/dl)※		Estradiol (pg/ml)§		17-OHP (nmol/L)▲	
		
		before tamoxifene	after tamoxifene	before tamoxifene	after tamoxifene	before tamoxifene	after tamoxifene	before tamoxifene	after tamoxifene	before tamoxifene	after tamoxifene
1	35	7.0	8.2	8.2	7.8	574.6	582.4	27.0	26.6	3.0	3.3
2	70	3.2	4.0	6.6	7.3	384.2	375.6	43.8	44.2	4.5	3.9
3	76	8.5	7.5	3.0	4.0	356.6	366.1	34.9	35.0	4.2	4.4
4	75	5.9	6.2	4.6	4.5	385.1	392.4	51.0	49.3	1.6	2.0
5	54	11.0	9.4	3.5	5.0	369.5	350.2	29.3	31.2	3.8	3.1
6☆	49	9.3	NA	5.4	NA	381.4	NA	71.2	NA	4.6	NA

*Normal value 1.5--12.5IU/L #Normal value 1.7--8.6IU/L ※Normal value 241.0-847.0 ng/dl

§Normal value 11.6-41.2 pg/ml ▲Normal value 0.6-5.4 nmol/L NA: not assayed.

☆The patient suffered from hepatitis B and tamoxifene was not given to him.

Abbreviations: FSH, follicle-stimulating hormone; LH, luteinizing hormone; 17-OHP, 17-hydroxyprogesterone.

During the follow-up one patient (49 years) died at 16 months after the beginning of imatinib mesylate treatment due to large burden of liver metastases. His overall survival was 35 months and progression free survival was 12 months. The other five patients were in good condition. The breast lesions completely disappeared in two patients and decreased in size in three.

## Discussion

Gynecomastia is defined as enlargement of the glandular tissue in the male breast. It is often found incidentally and ignored. In contrast, symptomatic gynecomastia is less common. The lesion is usually bilateral, but may present with unilateral mass, which is firm, mobile and disc-like, centrally located under the nipple-areola complex [5]. On palpation it is painful and tender. The pathologic findings of gynecomastia include hyperplasia of ductal epithelium, inflammatory cells infiltration of the periductal stroma and increased alveolar fat [6]. The pathophysiological process of gynecomastia includes imbalance between free estrogen and androgens acting in the breast tissue. This imbalance may occur through multiple mechanisms. Elevation of serum estrogen may result from estrogen secreting tumor, such as testicular or adrenocortical tumors. It may also be caused by an increased extra-gonadal conversion of androgen to estrogen by tissue aromatase [7]. Imbalance between testosterone and estrogen is caused by some kinds of sex hormone-binding globulin, which may be present in hyperthyroidism, chronic liver diseases and the use of medications, such as spironolactone. Spironolactone can block androgen production, block androgens from binding to their receptors, and increase both total and free estrogen levels [8,9]. During the course of imatinib mesylate treatment, there were three GIST patients who had received low dose spironolactone (20 mg daily) occasionally. However, gynecomastia did not occur in these three patients.

Gambacorti-Passerini et al measured hormone concentration in 38 men receiving imatinib for chronic leukemia at baseline and during treatment. Seven cases of gynecomastia were noted (18%) [2]. The incidence of gynecomastia is about 10% in our cohort patitents, which seems higher than we expected in clinical practice. The first reason we speculated may be that the patient felt shy and was reluctant to report his discomfort to physician. The second reason may be that the physicians apt to ignore this abnormality during regular physic examination On the contrary, we surgeons may be more sensitive to the changes of surgical condition.

Receptor tyrosine kinases c-Kit and platelet-derived growth-factor receptors alpha (PDGFRa) are expressed in the testis. They are believed to be involved in the bio-synthetic process of testosterone. Imatinib mesylate inhibits both c-Kit and PDGFRa and thus the production of testosterone may be decreased during its administration. Second-generation TKIs, such dasatinib and nilotinib, are multi-target inhibitor, including receptor tyrosine kinases c-Kit and PDGFRa. In fact, dasatinib exerts a more potent inhibitory action on c-Kit and PDGFRa [10]. Caocci et al reported a male patient suffered gynecomastia after treatment with dasatinib for CML [4].

In comparison of hormone concentration before and during imatinib treatment Gambacorti-Passerini et al found that patients who developed gynecomastia had more reduction in free testosterone concentration than those who did not [2]. Kim et al reported a patient who developed gynecomastia when treated with imatinib for GIST and demonstrated a lower serum concentration of testosterone and decreased libido. The patient concurrently had hydrocele of the testis which was improved when imatinib was discontinued, but the lesion recurred when the treatment was restarted. The authors concluded that inhibition of c-Kit and PDGFRa is the cause of the lesions described [3]. Animal studies demonstrated that PDGF signaling may constitute a common mechanism in the control of multiple steroidogenic lineages, and the c-Kit gene plays a fundamental role during the establishment, the maintenance and the function of germ cells [11-13].

In this study we did not find a decrease in free testosterone level in our patients with gynecomastia. There was no significant difference of free testosterone level in patients with or without gynecomastia who were taking regular dose (400 mg daily) of imatinib. In addition, our patients with gynecomastia do not have changes in their sexual behavior except one who was 76 years old with decreased libido. The factor of aging cannot be ruled out in this case. The increase in estrogen levels in half of our patients seems to suggest that an imbalance of sex-hormones may be the cause of gynecomastia development.

Generally there are three options for the treatment of gynecomastia, including radiation, surgery and medical therapy. Medicines including androgens, antiestrogens, aromatase inhibitors, danazol have been used to treat gynecomastia [14]. Our patients with gynecomastia had a good response to tamoxifene administration. Since imatinib metabolized in vivo mainly through CYP3A4, and tamoxifene is a substrate of CYP3A4. Their concomitant use may enhance the effect of tamoxifene [15].

## Conclusions

Although there was no special medications except imatinib taken by our patients before the develop of gynecomastia, the small sample size in this study prevents us to reach any conclusion at this point. So far there has not been a single publication regarding gynecomastia in the toxicity profile studies of imatinib mesylate. Given the fact that thousands of patients with chronic myeloid leukemia or GIST have received imatinib mesylate, it is unlikely that the breast lesions have been unnoticed. The purpose of the present report is to call for attention to this uncommon event, further study is warranted.

## Competing interests

Nothing to declare. No grant or commercial support was obtained for this study.

## Authors' contributions

HLL carried out data gathering and writing the study. GQL was involved in the patients care. ZSY was involved conceptualizing. All authors read and approved the final manuscript.

## Pre-publication history

The pre-publication history for this paper can be accessed here:

http://www.biomedcentral.com/1471-230X/11/116/prepub
